# IFN‐γ enhances the therapeutic efficacy of MSCs‐derived exosome via miR‐126‐3p in diabetic wound healing by targeting SPRED1

**DOI:** 10.1111/1753-0407.13465

**Published:** 2023-08-30

**Authors:** Wen Lu, Xuan Du, Shengyi Zou, Qionglei Fang, Mengjiao Wu, Huijuan Li, Bimin Shi

**Affiliations:** ^1^ Department of Endocrinology and Metabolism The First Affiliated Hospital of Soochow University Suzhou China

**Keywords:** diabetic wound healing, exosomes, interferon‐γ, mesenchymal stem cells, miR‐126‐3p, SPRED1

## Abstract

**Background and Aims:**

The traditional treatment of diabetic wounds is unsatisfactory. Exosomes isolated from bone marrow mesenchymal stem cells (BMSCs) promote the healing of diabetic wounds. However, whether the exosomes secreted by interferon (IFN)‐γ‐pretreated BMSCs have an enhanced therapeutic effect on diabetic wound healing and the relevant mechanisms remain unclear.

**Methods:**

In this study, we isolated exosomes from the corresponding supernatants of BMSCs with (IExos) or without IFN‐γ treatment (NExos). Human umbilical vein endothelial cells (HUVECs) were used to investigate the proliferation, migration, and tube formation under different treatments in vitro. Diabetic mice were induced by intraperitoneal administration of streptozotocin, and a circular full‐thickness dermal defect was then made on the back of each mouse, followed by a multisite subcutaneous injection of phosphate buffered saline or exosomes. Hematoxylin–eosin (H&E) staining, Masson's trichrome staining, and histological analysis were performed to assess the speed and quality of wound healing.

**Results:**

NExos treatment accelerated the healing of diabetic wounds by promoting angiogenesis in vivo and in vitro, and IExos exhibited superior therapeutic efficiency. MicroRNA (miR)‐126‐3p was significantly increased in IExos, and exosomal miR‐126‐3p promoted angiogenesis and diabetic wound healing via its transfer to HUVECs. miR‐126‐3p regulates SPRED1 by directly targeting the 3′‐UTR. Mechanistically, IFN‐γ‐pretreated BMSCs secreted miR‐126‐3p‐enriched exosomes, which enhanced the function of HUVECs and promoted angiogenesis via the SPRED1/Ras/Erk pathway.

**Conclusion:**

Exosomal miR‐126‐3p secreted from IFN‐γ‐pretreated BMSCs exhibited higher therapeutic efficacy than NExos in diabetic wound healing by promoting angiogenesis via the SPRED1/Ras/Erk axis.

## Graphical abstract

Highlights

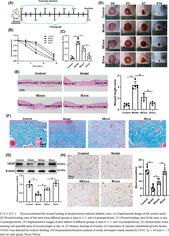

## 1 BACKGROUND

Diabetes mellitus affects over 422 million patients worldwide and up to 20% have developed impaired cutaneous wound healing or chronic ulcers.^1^ Impaired wound healing in diabetes often leads to nonunion or slow healing, which results in a significant downshift in patients' quality of life.^2^ However, traditional treatment of diabetic wounds, which mainly relies on dressing, negative pressure, hyperbaric oxygen, etc., is not satisfactory.^3^ Therefore, there is an urgent need to develop new potential materials or therapies for diabetic wound healing.

Therapies based on mesenchymal stem cells (MSCs) have recently attracted much interest.^4^ However, worries about immune‐mediated rejection, potential malignant transformation, and limited activity of transplanted cells make the practical application of MSCs problematic.^5^ Exosomes, which are extracellular vesicles with a diameter of 30–200 nm, are particles released by cells that carry bioactive molecules. Compared with cell‐based therapies, exosomes have several attractive advantages, including negligible rejection complications, good compatibility, non‐oncogenicity, and high stability.^6^ Recently, several studies have reported that exosomes isolated from MSCs can promote diabetic wound healing.^7^ Because the characteristics of exosomes depend on the status of MSCs, the therapeutic effects of exosomes are usually enhanced when the original MSCs are primed by cytokines such as interferon (IFN)‐γ and tumor necrosis factor alpha (TNF‐α).^8^ However, whether the exosomes secreted by MSCs after IFN‐γ pretreatment have an enhanced therapeutic effect on wound healing under high glucose (HG) conditions and the relevant mechanisms remain unclear.

Wound healing is a complex and dynamic process involving various components, and angiogenesis is one of the most important factors influencing wound repair.^9^ It determines the transport of nutrients and oxygen to the wound sites, which in turn affects fibroblast growth, collagen production, and reepithelialization, allowing the wound to enter the resolution phase of healing.^10^ Patients with diabetes experience slow wound healing owing to impaired angiogenesis. Enhancing angiogenesis has been linked to improved wound healing, and promoting neo‐angiogenesis with vascular endothelial growth factors (VEGF) has been successfully demonstrated to be therapeutic.^11^ Various studies have reported that regenerative medicine based on exosome use is promising for angiogenesis promotion. For example, Hu et al found that exosomal microRNA (miR)‐21‐5p isolated from bone marrow MSCs (BMSCs) increased angiogenesis in ischemic stroke mice.^12^ It has also been reported that exosomal lncRNA KLF3‐AS1 extracted from BMSCs can accelerate the healing of diabetic wounds via angiogenesis promotion.^13^ In the present study, we sought to determine whether exosomes derived from IFN‐γ‐pretreated BMSCs could improve the function of human umbilical vein endothelial cells (HUVECs) and promote angiogenesis in diabetic wound healing. The molecular mechanisms involved in the healing of diabetic wounds were also investigated.

## 2 METHODS

### 2.1 Cell culture

Human BMSCs were purchased from the American Type Culture Collection (ATCC, Manassas, VA, USA) and HUVECs were obtained from the Cell Bank of the Chinese Academy of Science (Shanghai, China). All cell lines were maintained at 37°C with 5% CO_2_ in a humidified chamber. Modified Eagle's medium (MEM; Hyclone, Logan, UT, USA) containing 10% fetal bovine serum (FBS) and 1% penicillin/streptomycin was used to cultivate the BMSCs. To validate the effect of IFN‐γ priming on BMSCs, when BMSCs reached 80% confluence, exosome‐free media supplemented with or without 25 ng/mL IFN‐γ was used to cultivate the cells for 48 h, and the conditioned medium (CM) was collected to extract exosomes (IExos and NExos). HUVECs were maintained in Dulbecco's MEM (Hyclone) supplemented with 10% FBS and 1% penicillin/streptomycin. To simulate hyperglycemic conditions, HUVECs were treated with HG (33 mM glucose) (Sigma‐Aldrich, St. Louis, MO, USA). The low‐glucose group (LG, 5.56 mM glucose + 27.44 mM mannitol) was used as a control. Exosomes (50 μg/mL) derived from BMSC‐CM under different conditions were co‐cultured with HUVECs.

### 2.2 Characterization of BMSCs

BMSCs were cultured in OriCell osteogenic and adipogenic differentiation media (Cyagen, Guangzhou, China). Alizarin Red and Oil Red O were used to identify osteogenic and adipogenic differentiation, respectively. To confirm the surface markers of BMSCs, the cells were stained with allophycocyanin‐conjugated antibodies (human anti‐CD34, anti‐CD45, and anti‐CD105) (BioLegend, San Diego, CA, USA). The corresponding isotype‐matched IgG antibodies (BioLegend) were used as negative controls. A flow cytometer (FACSCalibur; BD Biosciences, Franklin Lakes, NJ, USA) was used to detect fluorescence signals, and FlowJo software (BD Biosciences) was used to analyze the results.

### 2.3 Isolation and identification of exosomes

When the BMSCs attained 80% confluence, the culture medium was replaced with exosome‐free FBS and maintained with or without 25 ng/mL IFNγ for 48 h. The culture medium was collected and exosomes were extracted according to a previously reported method.^14^ Briefly, the collected medium was centrifuged at 300 × g for 10 min, 2000 × g for 20 min, 10 000 × g for 30 min, and 100 000 × g for 70 min. Finally, the precipitated pellets were centrifuged again at 100000 g for 70 min to yield purified exosomes and stored at −80°C for later use.

Transmission electron microscopy (TEM, Tecnai G2) was employed to capture the morphology of exosomes isolated from BMSCs treated with or without IFNγ. A nanoparticle tracking system (Nanosight Ltd., Navato, CA, USA) was used to determine the dimensions of NExos and IExos. Exosomal surface markers (TSG101, CD9, and CD63) were identified using western blotting.

### 2.4 Exosome uptake by HUVECs

Fluorescent dye was used to identify the exosomes according to the manufacturer's instructions. Briefly, exosomes were cultured for 15 min in a 2 mg/mL Dilute (Dil) solution (Sigma–Aldrich). The incubation solution was centrifuged at 100 000 × g for 60 min at 4°C to eliminate the excess Dil and get the labeled exosomes. These Dil‐labeled exosomes were co‐cultured with HUVECs for 12 h before harvest. The cells were stained with 4,6‐diamidino‐2‐phenylindole (DAPI; Thermo Fisher Scientific, Waltham, MA, USA). A confocal imaging system (Zeiss LSM880) was used to detect the uptake of Dil‐labeled NExos and IExos by HUVECs, and the fluorescence of Dil was analyzed using ZEN lite software between the two groups.

### 2.5 Cell counting kit‐8 assay

A Cell Counting Kit‐8 (CCK‐8) assay (Dojindo, Japan) was performed to assess cell proliferation in accordance with the manufacturer's instructions. Briefly, endothelial cells were seeded into 96‐well plates (2 × 10^3^ cells/well) and cultured for 3 days under various conditions. CCK‐8 solution (10 μL) diluted in 90 μL medium was added to each well every 24 h and incubated for 1 h at 37°C. The optical density (OD) was measured using a microplate reader (BioTek, Winooski, VT, USA) at 450 nm to assess cell proliferation.

### 2.6 5‐ethynyl‐2′‐deoxyuridine assay

The 5‐ethynyl‐2′‐deoxyuridine (EdU) assay (Ribobio, China) was used to detect the proliferation of HUVECs. Endothelial cells were seeded into 96‐well plates (1 × 10^4^ cells per well) and were then co‐cultured with EdU (200 μL; 50 mM) for another 2 h at 37°C. After the cells were fixed and permeabilized, ApolloR reaction cocktail was added, and Hoechst 33342 was used to visualize the nuclei. Images were captured using a Nikon TI‐DH light microscope (Nikon Corporation, Tokyo, Japan).

### 2.7 Scratch wound assay

HUVECs were seeded into six‐well plates under different culture conditions until they reached 100% confluence. A 200 μL pipette tip was used to scratch the cell layer at the center of the wells to create a wound. The wounds were then carefully washed with phosphate buffered saline (PBS) and serum‐free medium was added to enable cell migration. Images were captured 0 and 24 h after scratching. To assess the cell migration capability, the migration ratio was defined as the migrated area/initial wound area.

### 2.8 Tube formation assay

Each well of a 96‐well dish was covered with 50 μL of Matrigel (ABW, China), which was solidified for 45 min before use. Precultured HUVECs were then resuspended and seeded in 96‐well plates (1.5 × 10^4^ cells/well). Ten hours later, the HUVECs were stained using the Calcein‐AM dye (MCE, USA) at a concentration of 6.25 g/mL for 30 min at 37°C. Capillary‐like structures were photographed using a Nikon TI‐DH light microscope (Nikon). The total tube length from three randomly chosen fields was analyzed using Image J to assess angiogenesis.

### 2.9 Quantitative reverse transcription‐polymerase chain reaction (qRT‐PCR)

TRIzol reagent (Invitrogen, Waltham, MA, USA) was used to extract total RNA from the cells, and total exosomal miRNAs were extracted using the SeraMir Exosome RNA Purification Kit (System Biosciences, Mountain View, CA, USA). RevertAid First‐Strand cDNA Synthesis Kit (Takara, Dalian, China) or Bulge‐Loop miRNA qRT‐PCR Starter Kit (RiboBio) was used to synthesize cDNA. qRT‐PCR was performed on an ABI 7300 instrument (Applied Biosystems, Waltham, MA, USA) using SYBR Premix Ex Taq (Takara). The specific primers used in this study are listed in Table S1. β‐actin or U6 was used as an endogenous control.

### 2.10 Western blot analysis

Proteins from exosomes and endothelial cells were collected and extracted using RIPA lysis buffer (Beyotime Institute of Biotechnology, Shanghai, China). The lysate was centrifuged at 12 000 rpm to obtain protein extracts. The protein concentration was measured using a bicinchoninic acid protein assay kit (Beyotime). Next, 15 μg of protein was loaded, separated using sodium dodecyl sulfate–polyacrylamide gel electrophoresis, and transferred to polyvinylidene difluoride membranes (Millipore, Mississauga, Canada). The membranes were incubated with the primary antibodies overnight at 4°C after being blocked with 5% nonfat milk for 1 h. Then, they were incubated with the appropriate secondary antibodies at room temperature for 1 h. The following primary antibodies were used: anti‐CD9, anti‐CD63, and anti‐TSG101 (1:1000; Abclonal, Woburn, MA, USA); anti‐SPRED1, anti‐Ras, and anti‐p/t‐Raf (1:1000; Abcam, Cambridge, UK); anti‐p/t‐MEK1/2 and anti‐p/t‐ERK1/2 (1:1000; Bioworld, Nanjing, China); and anti‐β‐actin (1:10000; Proteintech, Rosemont, IL, USA). The Tanon‐5200 Chemiluminescent Imaging System (Tanon Science & Technology, China) was used to detect the protein bands, after which the grayscale value was calculated using Image J software.

### 2.11 miRNA target prediction

Four online databases, TargetScan (http://www.targetscan.org), MiRDB (http://mirdb.org/), mirDIP (http://ophid.utoronto.ca/mirDIP), and DIANA (http://diana.imis.athenainnovation.gr/DianaTools), were used to predict the putative mRNA targets of miR‐126‐3p. The top 19 genes in each database were used to determine the intersection.

### 2.12 Luciferase reporter assay

The wild‐type (WT) or mutant (MUT) sequence containing the binding site of the 3′‐UTR of SPRED1 was inserted into the pGL3‐REPORT luciferase vectors (SPRED1‐WT and SPRED1‐MUT) (GeneScript, Nanjing, China). HUVECs were first transfected with these vectors, seeded into 96‐well plates, and co‐transfected with miR‐NC or miR‐126‐3p inhibitor (GenePharma, Shanghai, China). A Dual‐Luciferase kit (Promega, Madison, WI, USA) was used to measure luciferase activity according to the manufacturer's instructions.

### 2.13 Animal model

Male C57BL/6J mice (8 weeks old) were purchased from the Model Animal Research Center of Nanjing University (Nanjing, China) and kept in a specified pathogen‐free environment with a 12‐h light/dark cycle. This animal study was approved by the Animal Ethics Committee of Soochow University (Approval Number: No. 250 [2019]). Diabetes was induced as described previously.^13^, ^15^ Briefly, the mice were intraperitoneally administered streptozotocin (STZ) (40 mg/kg, Sigma–Aldrich) for 5 days. These mice were fed a high‐fat diet for 6 weeks to create a stable animal model of diabetes mellitus. Blood glucose was then measured, and those with fasting blood glucose level over 16.7 mM for two successive measurements were used for the following wound healing study.

Pentobarbital sodium was administered intraperitoneally to diabetic mice (50 mg/kg; Sigma–Aldrich), and a 10‐mm‐diameter circular full‐thickness dermal defect was made on the back of each animal. On days 0, 3, 5, 7, 9, and 11 following wound development (*n* = 6), the mice underwent multisite subcutaneous injections (at least six locations) of 100 μL PBS or 100 μL exosomes (100 μg exosomes in 100 μL PBS) around the wounds. Normal mice (Ctrl) fed a normal diet and water underwent identical wound procedures. The wounds were covered with an occlusive dressing (Tegaderm; 3 M, St. Paul, MN, USA) and imaged on days 0, 3, 7, and 14. To compare the healing speed between the different groups, the wound healing ratio was defined as the remaining wound area/initial wound area.

### 2.14 Histological analysis

The mice were sacrificed on day 14, and the wound samples were treated with 4% paraformaldehyde solution before being embedded in paraffin. The wound specimens were then sliced into 5‐μm‐thick sections. Antigen retrieval was performed, and the sections were then blocked and incubated with the primary antibody against cluster of differentiation 31 (CD31; 1:2000; Abcam) overnight at 4°C before treatment with secondary antibody and ABC complex. Finally, the sections were stained with DAB substrate, and the stained images were captured using a Nikon TI‐DH light microscope (Nikon). H&E and Masson's trichrome staining were conducted following established protocols.

### 2.15 Immunofluorescence analysis

For immunofluorescence staining, 5‐μm‐thick sections underwent xylol deparaffinization, rehydration, and antigen retrieval. The specimens were blocked using blocking buffer and permeabilized with 0.1% Triton X‐100. The slides were then treated with the CD31 and α‐smooth muscle actin (1:300, Abcam) primary antibodies overnight at 4°C before being incubated with Cy5‐ and FITC‐conjugated secondary antibodies (1:400; Sigma–Aldrich) for 60 min at 25°C in the dark. Finally, nuclei were stained with DAPI after incubation with 0.1% DAPI solution for 10 min at 20°C. Images were captured using a confocal laser‐scanning microscope (Zeiss LSM880; Zeiss, Oberkochen, Germany).

### 2.16 Statistical analyses

All data are expressed as mean ± SD. Student's *t* test was used to analyze the statistical differences between two groups, and one‐way analysis of variance was used to evaluate differences between multiple groups. All analyses were performed using GraphPad Prism 8.0 (GraphPad, San Diego, CA, USA), and IBM SPSS 21.0 (SPSS Inc., Chicago, IL, USA). Statistical significance was set at *p* < .05.

## 3 RESULTS

### 3.1 Identification of BMSCs

To verify the presence of BMSCs, the morphology of the cells was captured as described, which exhibited a spindle‐like shape (Figure S1A). These cells also showed osteogenic and adipogenic differentiation, as detected by Alizarin Red and Oil Red O staining, respectively (Figure S1B). Flow cytometry analysis was used to identify BMSCs, and the isolated cells showed positive expression of CD90 and CD44, but negative for CD34 (Figure S1C).

### 3.2 Characterization of BMSCs‐derived exosomes

**FIGURE 1:**
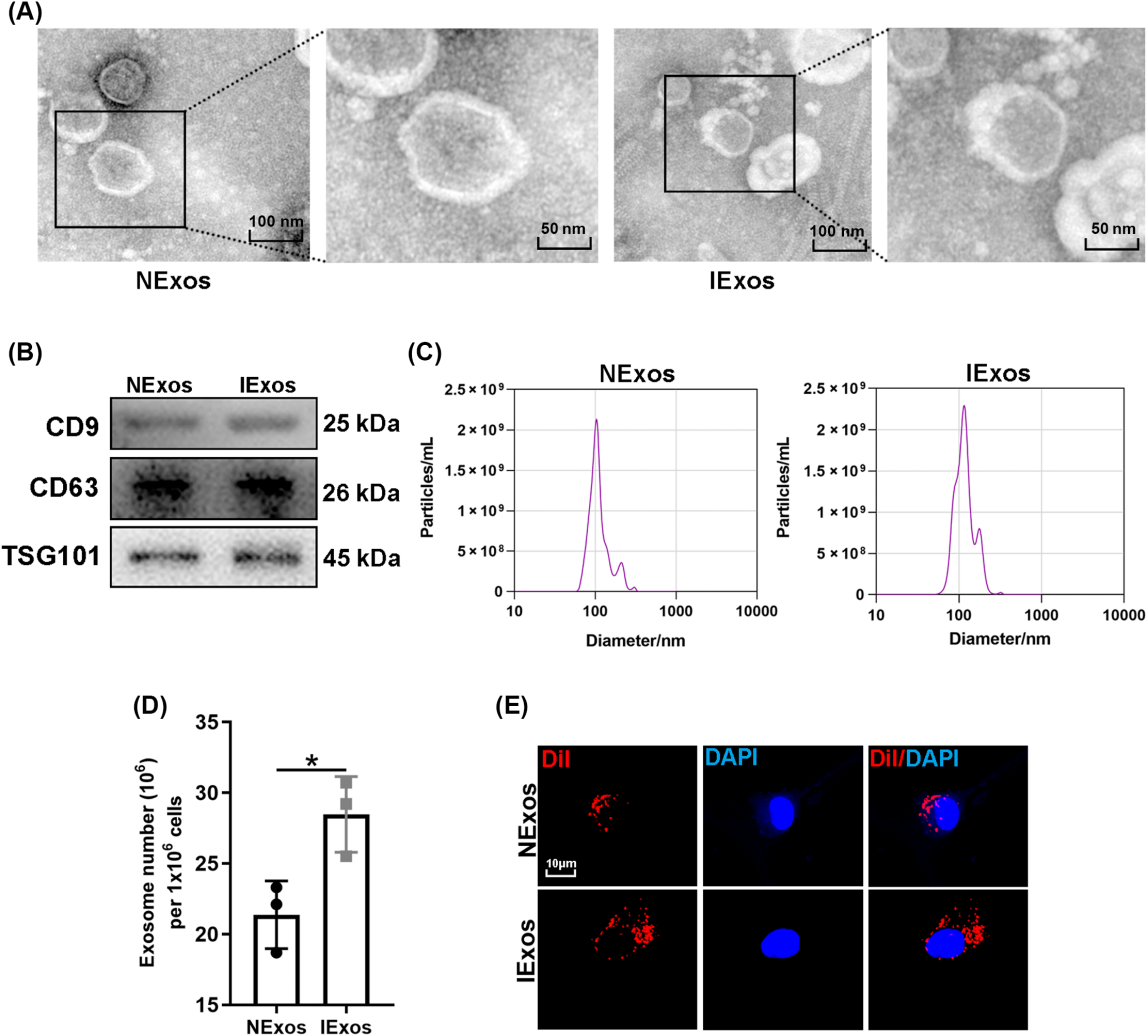
The characterization of bone marrow mesenchymal stem cells (BMSC)‐derived exosomes. (A) The morphology of NExos and IExos was examined by transmission electron microscopy (TEM). (B) The specific surface markers (CD9, CD63, and TSG101) of NExos and IExos were assessed by Western blotting. (C) The size concentration of NExos and IExos were detected via nanoparticle tracking analysis (NTA) (D) Exosome number per million cells was calculated to estimate the yield of exosomes secreted by BMSCs after interferon (IFN)‐γ treatment. (E) Dil‐labeled NExos and HExos were internalized by human umbilical vein endothelial cells (HUVECs). (F) The fluorescence intensity of the dye‐labeled internalized exosomes were quantified between the two groups. *p < .05 and *n* = 3 biological replicates. DAPI, 4,6‐diamidino‐2‐phenylindole; IExos, exosomes isolated from the supernatants of BMSCs with IFN‐γ treatment; NExos, exosomes isolated from the supernatants of BMSCs without IFN‐γ treatment.

CM of BMSCs with or without IFN‐γ treatment was collected, and the corresponding exosomes (IExos and NExos) were isolated. To identify BMSC‐derived exosomes, the particles were first assessed by TEM, and both types of exosomes exhibited typical exosomal structures (Figure 1A). Exosomal surface markers such as CD9, CD63, and TSG101 were identified by western blotting of the isolated particles (Figure 1B). Moreover, the size distribution determined by nanoparticle tracking analysis (NTA) suggested that both Exos ranged from 50 to 200 nm. (Figure 1C). These results confirmed the successful extraction of exosomes and indicated that treatment with IFN‐γ did not significantly affect BMSC‐Exos in terms of morphology and particle size. IFN‐γ treatment has been reported to enhance the secretion of exosomes by human umbilical MSCs.^16^ We investigated whether the yield of exosomes from IFN‐γ‐treated BMSCs would be affected in this study. The number of exosomes per million cells indicated a higher yield secreted by BMSCs after IFN‐γ treatment (Figure 1D). To determine whether BMSC‐Exos could be internalized into HUVECs, exosomes labeled with Dil were co‐cultured with HUVECs for 12 h. The internalization of exosomes by HUVEC was detected 12 h later, and red dye was detected in endothelial cells in both groups, which suggested that both types of exosomes could be captured by HUVEC (Figure 1E).

### 3.3 IExos accelerated the healing of diabetic wound by inducing angiogenesis

**FIGURE 2:**
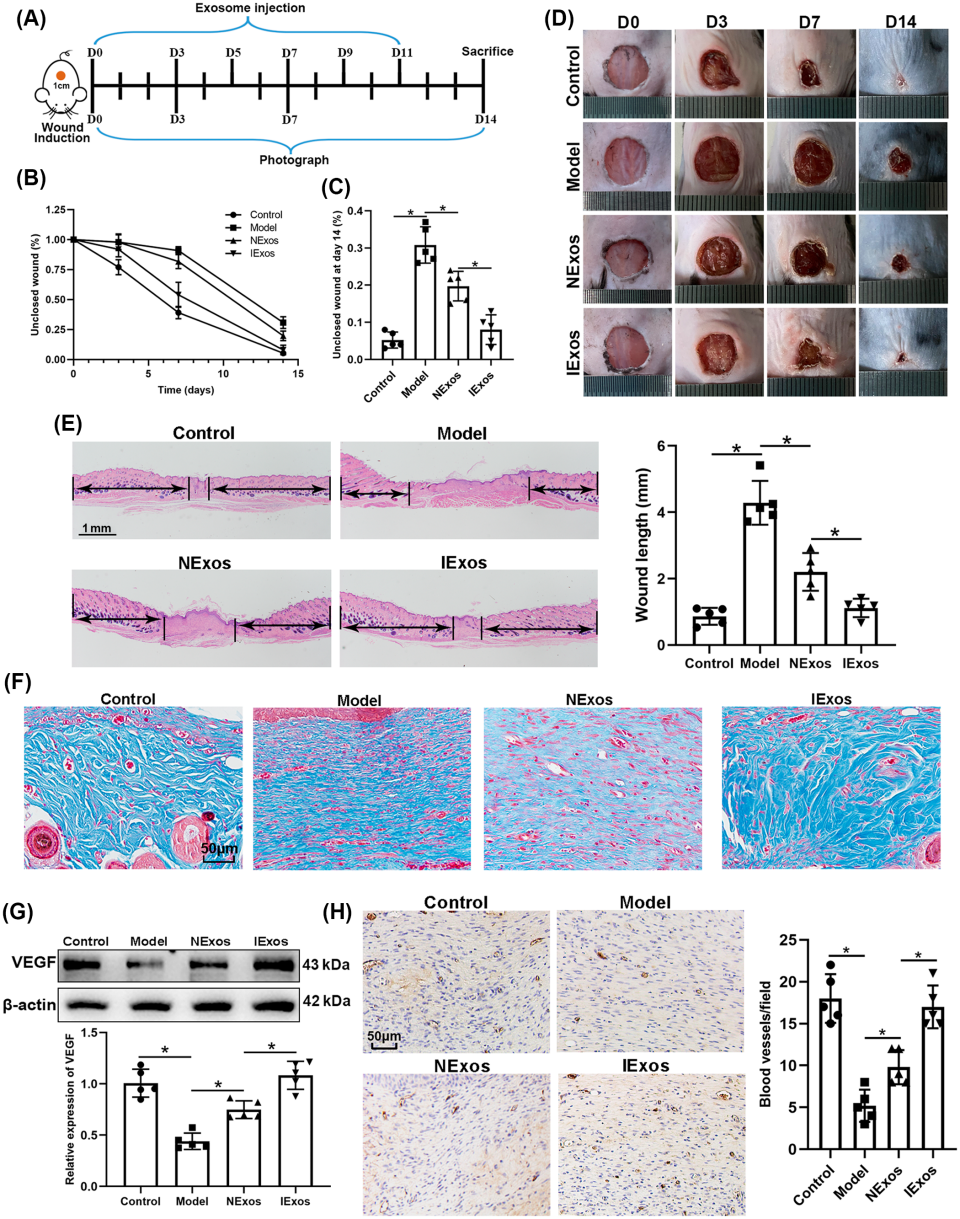
IExos accelerated the wound healing of streptozotocin‐induced diabetic mice. (A) Experimental design of the animal study. (B) Wound healing rates of the mice from different groups at days 0, 3, 7, and 14 postoperatively. (C) Wound healing rates of the mice at day 14 postoperatively. (D) Representative images of skin defects of different groups at days 0, 3, 7, and 14 postoperatively. (E) Hematoxylin–eosin staining and quantification of wound length at day 14. (F) Masson staining of wounds. (G) Expression of vascular endothelial growth factors (VEGF) was detected by western blotting. (H) Immunohistochemical analysis of newly developed vessels stained by CD31. *p < .05 and *n* = 5 mice in each group. IExos, exosomes isolated from the supernatants of BMSCs with IFN‐γ treatment; NExos, exosomes isolated from the supernatants of BMSCs without IFN‐γ treatment.

We investigated whether IExos could promote wound healing under HG conditions in vivo. In this study, diabetic mouse models were established by STZ treatment. At the end of the induction period, mice with fasting blood glucose level over 16.7 mM for two successive measurements were used for the subsequent wound healing study. A control group of mice was generated by injecting saline into mice at the same volume of STZ. The timescale for the injection of PBS/exosomes around the wounds is shown in Figure 2A. Briefly, full‐thickness skin defects were developed on the mice back and then they received subcutaneous injections of PBS (Ctrl group), PBS (Model group), NExos (NExos group), and IExos (IExos group) around the skin defects. Wound closure was monitored on days 0, 3, 7, and 14. The results showed that wound closure in both Exos groups was accelerated compared to that in the Model group, whereas IExos presented an enhanced therapeutic effect (Figure 2B–D). Additionally, compared with the Model group, the wounds of both Exos‐treated groups had better reepithelization, as reflected by more epithelial structures (Figure 2E). The results also showed that the wound length in the NExos and IExos groups was shorter than that in the Model group (Figure 2E). Masson staining also indicated the extracellular matrix remodeling ability of both Exos, as evidenced by thicker fibers and more extensive collagen deposition (Figure 2F). The therapeutic effect was further enhanced in the IExos group compared with that in the NExos group.

Patients with diabetes often experience slow wound healing due to impaired angiogenesis. VEGF induces angiogenesis^17^ and CD31 has been recognized as a marker of newly formed vessels.^18^ The results of this study showed that VEGF was upregulated in the NExos and IExos groups (Figure 2G), and the number of blood vessels increased in the Exos‐treated groups (Figure 2H), especially in the IExos group. Collectively, IExos treatment accelerates diabetic wound healing by promoting angiogenesis.

### 3.4 IExos promote proliferation, migration, and tube formation in HUVECs

**FIGURE 3:**
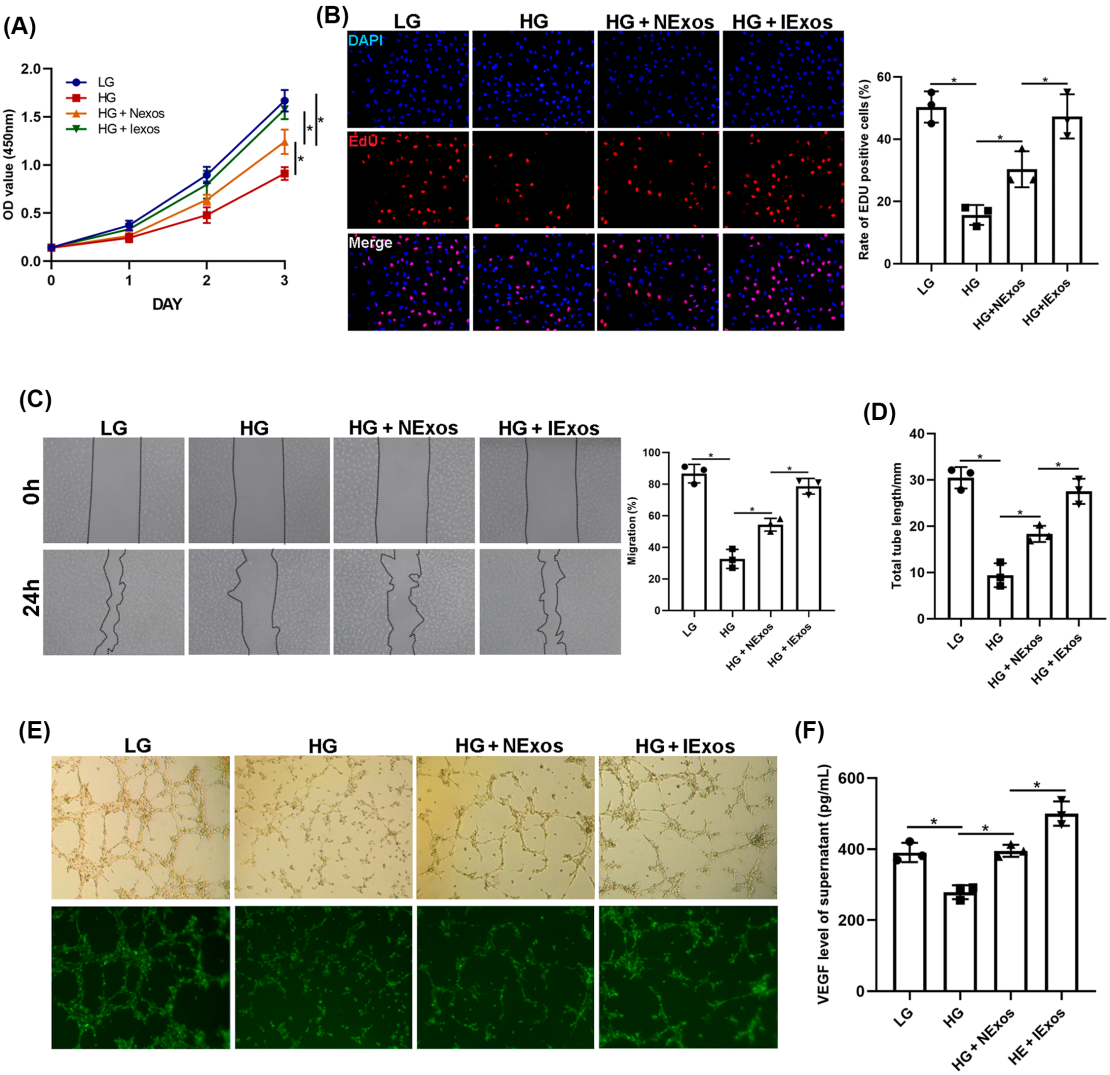
IExos promoted the function of human umbilical vein endothelial cells (HUVECs). (A) The cell counting kit‐8 (CCK8) and (B) 5‐ethynyl‐2′‐deoxyuridine (EdU) assay were conducted to detect the cell proliferation. (C) Wound healing assay was performed to detect the cell migration of endothelial cells. The tube formation tests were performed (E) and quantified (D) to investigate the pro‐angiogenic capacity of Exos. (F) vascular endothelial growth factors (VEGF) secretion by endothelial cells were assessed through ELISA. *p < .05 and *n* = 3 biological replicates. HG, high glucose; IExos, exosomes isolated from the supernatants of BMSCs with IFN‐γ treatment; LG, low glucose; NExos, exosomes isolated from the supernatants of BMSCs without IFN‐γ treatment; OD, optical density.

To verify the results, HUVECs were used to test the angiogenesis‐promoting effect of IExos in vitro. The HUVECs were divided into four groups: LG, HG, HG + NExos, and HG + IExos. To detect cell proliferation, CCK8 and EdU assays were conducted, and the results showed that cell viability was improved in both Exos groups, especially after IExos treatment (Figure 3A,B). Consistent with these results, migration of endothelial cells, as reflected by the wound healing assay, was significantly increased after co‐culture with Exos (Figure 3C). To investigate the proangiogenic capacity of IExos, tube formation tests were performed and showed that a longer total tube length could be detected in both Exos groups than in the HG group (Figure 3D,E). VEGF secretion by the endothelial cells was also assessed. NExos and IExos can both promote VEGF production by HUVECs, and IExos has a stronger secretory effect (Figure 3F). Collectively, these findings confirm the effect of IExos on the biological functions of HUVECs in vitro.

### 3.5 miR‐126‐3p is enriched in IExos and internalized by HUVECs via exosomes

**FIGURE 4:**
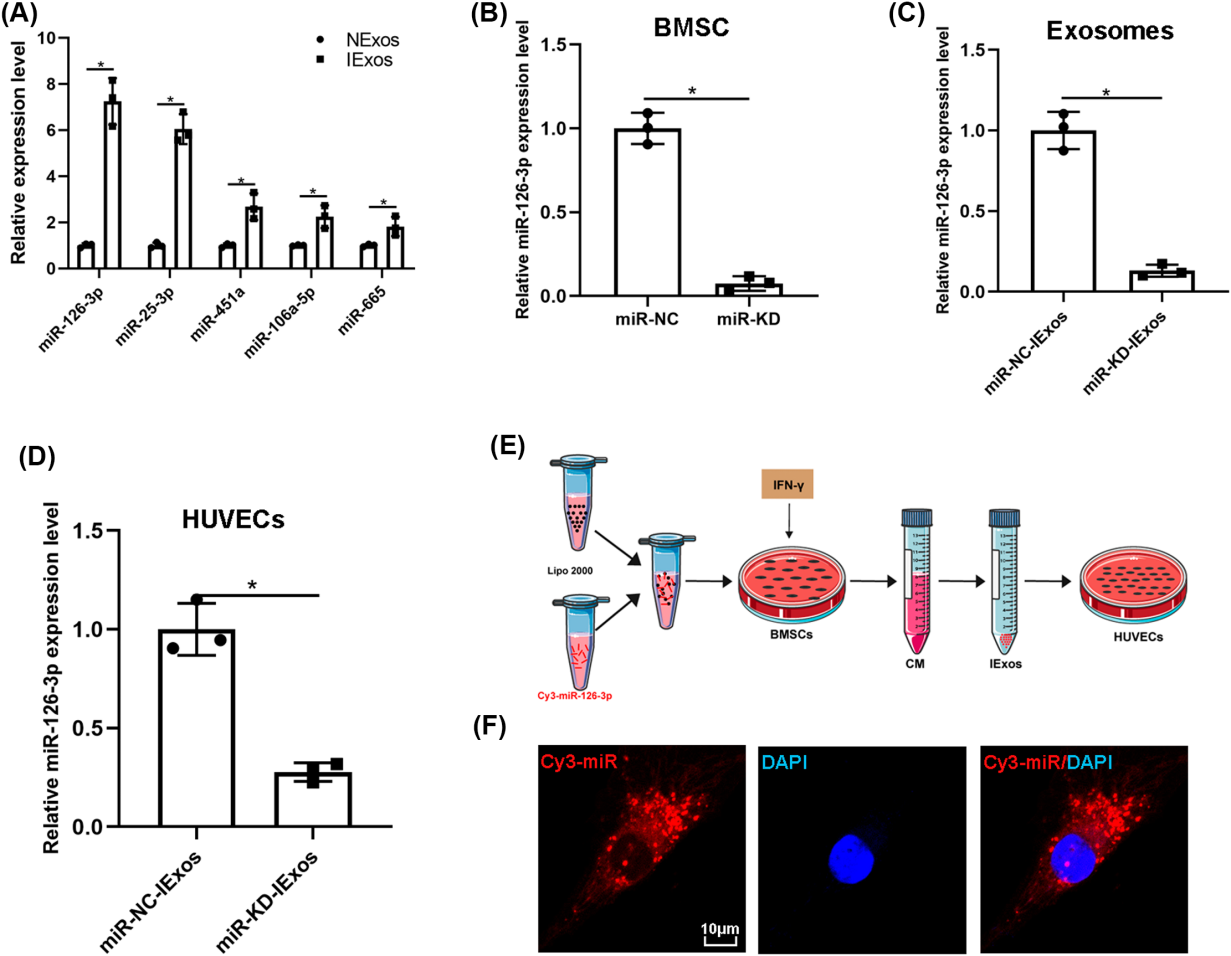
The miR‐126‐3p is upregulated in IExos and transferred to human umbilical vein endothelial cells (HUVECs) by exosomes. (A) The expression of exosomal miRNAs (miR‐25‐3p, miR‐106a‐5p, miR‐126‐3p, miR‐451a, and miR‐665) were confirmed through quantitative reverse transcription‐polymerase chain reaction (qRT‐PCR). U6 was used as an internal control. (B) The expression levels of miR‐126‐3p were detected by qRT–PCR to confirm the transfection efficiency. (C) The miR‐126‐3p expression levels were detected by qRT–PCR in exosomes of miR‐NC‐IExos and miR‐KD‐IExos. (D) After coculture with the miR‐NC‐IExos and miR‐KD‐IExos for 24 h, the miR‐126‐3p expression level was obviously decreased in HUVECs compared with the control group. (E) bone marrow mesenchymal stem cells (BMSCs) were transfected with Cy3‐labeled miR‐126‐3p mimics and the HUVECs were treated with according exosomes. (F) Cy3 immunofluorescence intensity confirmed the shuttling of exosomal Cy3‐miR‐126‐3p into recipient endothelial cells. *p < .05 and *n* = 3 biological replicates. CM, conditioned medium; DAPI, 4,6‐diamidino‐2‐phenylindole; IExos, exosomes isolated from the supernatants of BMSCs with IFN‐γ treatment; IFN‐γ, interferon‐gamma; miR, microRNA; NExos, exosomes isolated from the supernatants of BMSCs without IFN‐γ treatment.

Considering that various studies have reported that miRNAs are one of the main functional components of exosomes, we investigated whether miRNAs play a crucial role in IExos‐mediated angiogenesis. A previous study reported that IFN‐γ can induce a significant increase in the expression of five miRNAs, including miR‐25‐3p, miR‐106a‐5p, miR‐126‐3p, miR‐451a, and miR‐665.^19^ We confirmed the expression of miRNAs using qRT‐PCR and found that these miRNAs were also upregulated (Figure 4A). Considering that miR‐126‐3p was upregulated in IExos and was reported to promote exosome‐mediated angiogenesis under different conditions,^20^, ^21^, ^22^, ^23^, ^24^ we focused on miR‐126‐3p and investigated whether IExos could induce angiogenesis by transferring exosomal miR‐126‐3p. Thus, we knocked down miR‐126‐3p in BMCSs by transfecting them with an inhibitor of miR‐126‐3p (miR‐knockdown [KD]). Transfection efficiency was verified by qRT‐PCR, and the findings demonstrated that miR‐126‐3p expression was significantly decreased in the miR‐KD group compared to that in the miR‐NC group (Figure 4B). Exosomes were derived from miR‐NC‐BMSCs and miR‐KD‐BMSCs after treatment with IFN‐γ (miR‐NC‐IExos and miR‐KD‐IExos). As shown in Figure 4C, miR‐126‐3p expression was notably decreased in miR‐KD‐IExos. Furthermore, the expression level of miR‐126‐3p was significantly decreased in the target HUVECs treated with miR‐KD‐IExos (Figure 4D). To further confirm the transfer of exosomal miR‐126‐3p from BMSCs to HUVECs, exosomes were extracted from the medium of BMSCs transfected with Cy3 labeled miR‐126‐3p (Figure 4E). Consistent with the above results, red immunofluorescence was detected in the cytoplasm of target HUVECs, suggesting that miR‐126‐3p in BMSCs can be delivered to HUVECs via exosomes (Figure 4F). We wondered whether IFN‐γ pretreatment directly increase the expression of miR‐126‐3p or only enhance its enrichment in exosomes secreted by BMSCs. We detected the expression of miR‐126‐3p in BMSCs under different conditions and in corresponding exosomes. The expression level of miR‐126‐3p was increased in IFN‐γ‐primed BMSCs and the inhibitor of miR‐126‐3p was used to knock down the expression of miR‐126‐3p to normal levels (Figure S2A). Interestingly, as the expression of miR‐126‐3p in IFN‐γ‐primed BMSCs returned to normal, the expression of their exosomal miR‐126‐3p was also significantly downregulated (Figure S2B). We speculated that IFN‐γ pretreatment directly increase the expression of miR‐126‐3p in BMSCs, which resulted in the enrichment of exosomal miR‐126‐3p.

### 3.6 miR‐126‐3p KD suppresses IExos‐mediated proliferation, migration, and angiogenesis in vitro and in vivo

**FIGURE 5:**
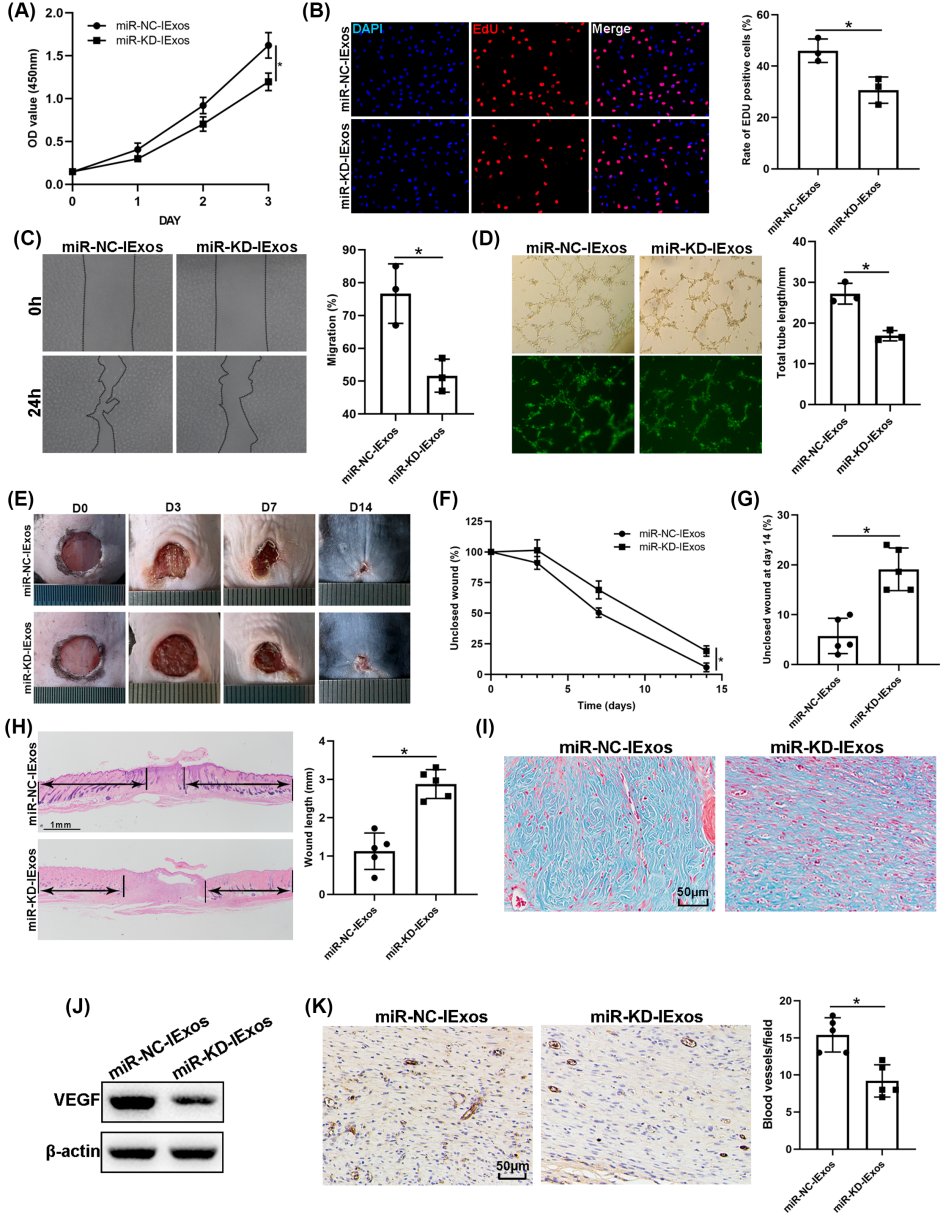
Knockdown of miR‐126‐3p inhibits IExos‐mediated proliferation, migration and angiogenesis in vitro and in vivo. (A) The cell counting kit‐8 (CCK8) and (B) cell counting kit‐8 (EdU) assays were used to detect the proliferation of human umbilical vein endothelial cells (HUVECs) in the miR‐NC‐IExos and miR‐KD‐IExos groups. (C) Wound healing assay was used to detect the migration of HUVECs. (D) Tube formation assay was used to detect the ability for vascular formation of HUVECs. (E) Representative images of skin defects of different groups at days 0, 3, 7, and 14 postoperatively. (F) Wound healing rates of the mice from different groups at days 0, 3, 7, and 14 postoperatively. (G) Wound healing rates of the mice at day 14 postoperatively. (H) Hematoxylin–eosin staining and quantification of wound length at day 14. (I) Masson staining of wounds. (J) Expression of vascular endothelial growth factors (VEGF) was detected by western blotting. (K) Immunohistochemical analysis of newly developed vessels stained by CD31. *p < .05, *n* = 3 biological replicates, and *n* = 5 mice in each group. IExos, exosomes isolated from the supernatants of BMSCs with IFN‐γ treatment; miR, microRNA; NExos, exosomes isolated from the supernatants of BMSCs without IFN‐γ treatment; OD, optical density.

Because miR‐126‐3p was upregulated in the exosomes derived from BMSCs pretreated with IFN‐γ, and that it can be transferred to target cells through exosomes, we then investigated the function of exosomal miR‐126‐3p in angiogenesis and wound healing. HUVECs were treated with miR‐NC‐IExos and miR‐KD‐IExos. The findings indicated that proliferation of HUVECs was inhibited in the miR‐KD‐IExos group compared with the control group, as reflected by CCK8 (Figure 5A) and EdU assays (Figure 5B). Migration assays also showed that miR‐KD‐IExos administration reduced the rate of scratch closure (Figure 5C). Moreover, the vascular formation ability of HUVECs was impaired by the miR‐KD‐IExos treatment (Figure 5D). These findings suggest that miR‐126‐3p KD suppresses HUVEC function in vitro.

Mice were then treated with miR‐NC‐IExos and miR‐KD‐IExos, as described previously. Wound closure was hindered in the miR‐KD‐IExos group compared with that in the Ctrl group **(**Figure 5E–G **)**, which was consistent with the wound length on day 14 (Figure 5H). Masson staining also showed that less collagen was deposited after miR‐KD‐IExos application (Figure 5I). VEGF was downregulated in the miR‐KD‐IExos group (Figure 5I) and the number of vessels marked by CD31 was lower after miR‐KD‐IExos treatment than in the miR‐NC‐IExos group (Figure 5J). Taken together, these results suggested that miR‐126‐3p plays a central role in IExos‐mediated angiogenesis and wound healing.

### 3.7 Exosomal miR‐126‐3p regulates SPRED1 by directly targeting the 3′–UTR

**FIGURE 6:**
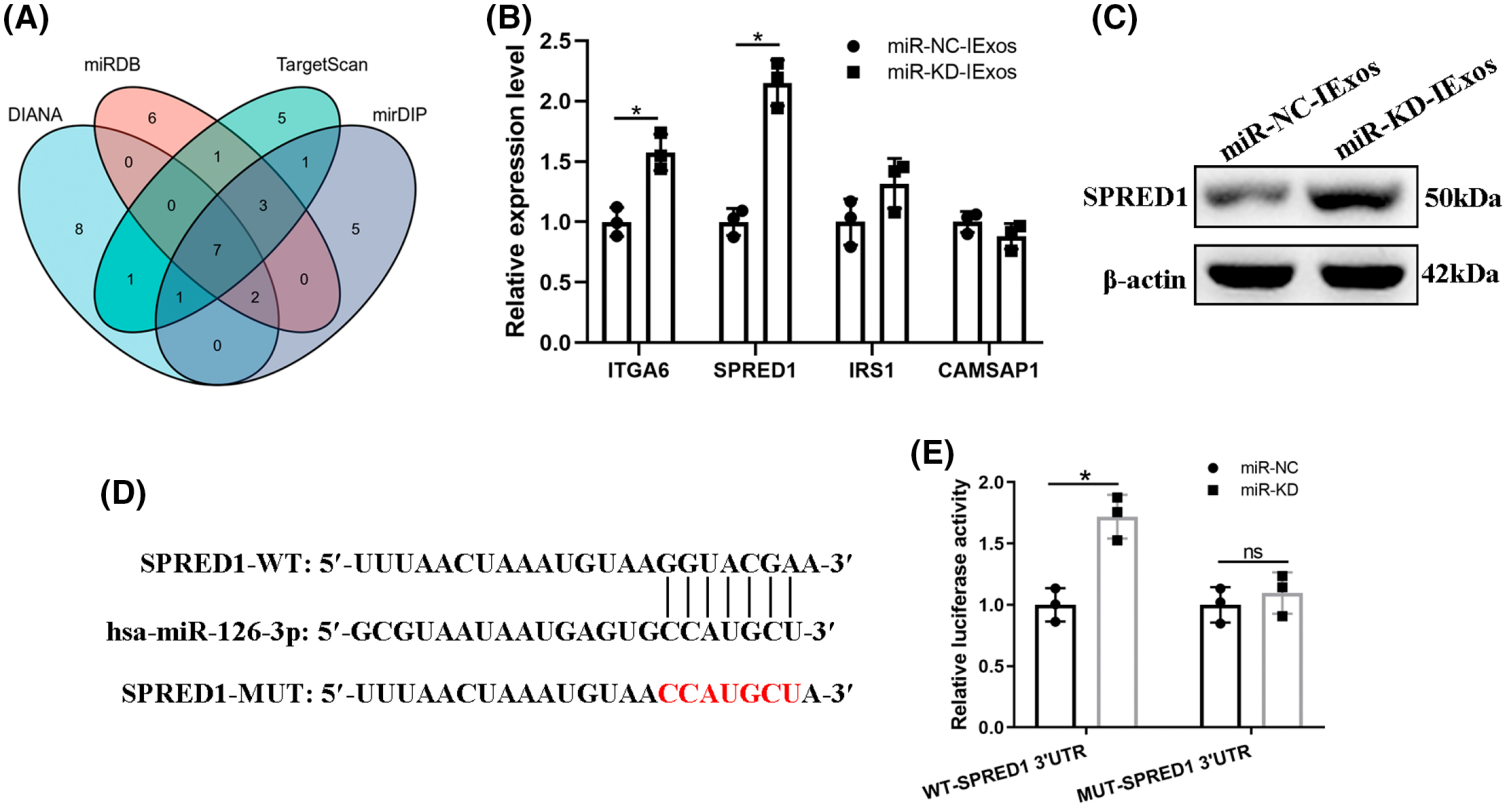
Exosomal miR‐126‐3p regulates SPRED1 by directly targeting the 3′–UTR. (A) Top 19 genes of each database (DIANA, miRDB, TargetScan, and mirDIP) were used to take the intersection and there were finally seven mRNAs left. (B) Quantitative reverse transcription‐polymerase chain reaction (qRT‐PCR) was used to determine the expression levels of the five selected mRNAs (ITGA6, SPRED1, IRS1, and CAMSAP1). (C) The protein expression level of SPRED1 was confirmed by western blotting. (D) Luciferase reporter assay was performed to verify that miR‐126‐3p can directly target the 3′UTR of SPRED1. (E) The relative luciferase activity was increased in the miR‐KD group compared with miR‐NC group in the wild‐type (WT) cells, but it was comparable in mutant (MUT) cells. *p < .05 and *n* = 3 biological replicates. miR, microRNA.

To further investigate the molecular mechanism of exosomal miR‐126‐3p in exosome‐mediated angiogenesis, four online databases were used to predict the mRNA targets of miR‐126‐3p: DIANA, miRDB, TargetScan, and mirDIP. As shown in Figure 6A, the top 19 genes from each database were used to take the intersection and there were finally seven mRNAs left (Figure 6A). Among them, five (ITGA6, SPRED1, IRS1, and CAMSAP1) have been reported to be associated with HUVECs and angiogenesis. qRT‐PCR was used to determine the expression levels of these mRNAs, and the results showed that SPRED1 was more strongly influenced by miR‐KD‐IExos than by miR‐NC‐IExos (Figure 6B **)**. Moreover, SPRED1 has been shown to play a negative role in endothelial cell proliferation, angiogenesis, and migration.^25^ Western blotting confirmed that SPRED1 was significantly upregulated by miR‐KD‐IExos (Figure 6C). To verify that miR‐126‐3p can directly target the 3′‐UTR of SPRED1, a luciferase reporter assay was performed (Figure 6D). The relative luciferase activity was increased in the miR‐KD group compared with the miR‐NC group in WT cells, but it was comparable in MUT cells (Figure 6E). Collectively, exosomal miR‐126‐3p can directly target SPRED1 and regulate its expression.

### 3.8 Exosomal miR‐126‐3p promotes HUVECs proliferation, migration, and angiogenesis by targeting SPRED1

**FIGURE 7:**
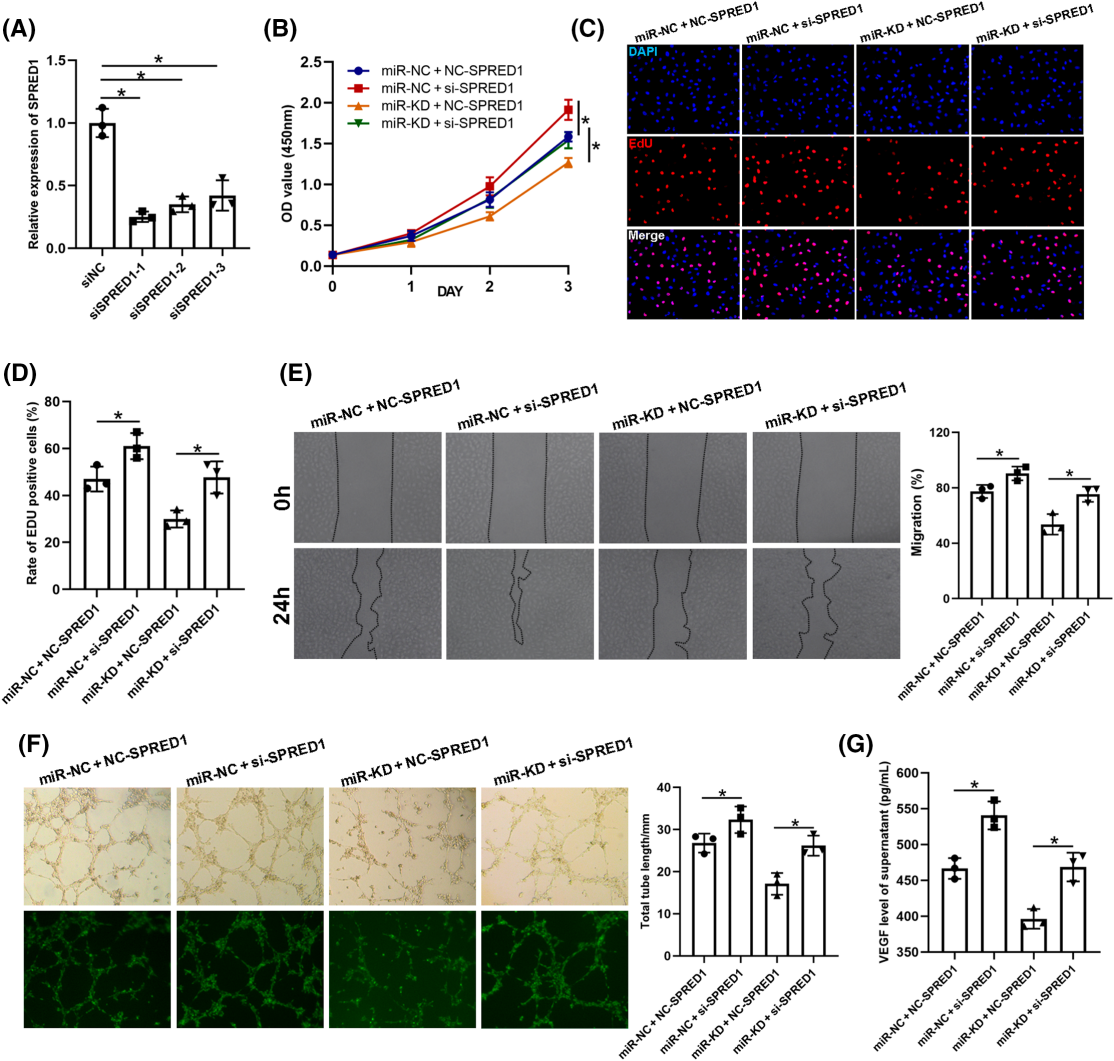
Exosomal miR‐126‐3p promotes human umbilical vein endothelial cells (HUVECs) proliferation, migration and angiogenesis by targeting SPRED1. (A) Quantitative reverse transcription‐polymerase chain reaction (qRT‐PCR) was used to confirm the knockdown efficiency of siSPRED1 in HUVECs. (B) The cell counting kit‐8 (CCK8) and (C and D) 5‐ethynyl‐2′‐deoxyuridine (EdU) assay were conducted to detect the cell proliferation of HUVECs after treatment with miR‐NC + NC‐SPRED1, miR‐NC + si‐SPRED1, miR‐KD + NC‐SPRED1, and miR‐KD + si‐SPRED1. (E) Wound healing assay was performed to detect the cell migration of endothelial cells. (F) The tube formation tests were performed to investigate the pro‐angiogenic capacity of HUVECs. (G) vascular endothelial growth factors (VEGF) secretion by endothelial cells were assessed through ELISA. *p < .05 and *n* = 3 biological replicates. miR, microRNA; OD, optical density.

Several rescue experiments were performed to verify the relationship between exosomal miR‐126‐3p and SPRED1. First, HUVECs were transfected with siNC and siSPRED1, and KD efficiency was confirmed by qRT‐PCR (Figure 7A). siSPRED1‐1 was used for further experiments, and the transfected cells were then treated with miR‐NC‐IExos and miR‐KD‐IExos. SPRED1 KD promoted the proliferation (Figure 7B–D), migration (Figure 7E), and angiogenesis of HUVECs (Figure 7F). Furthermore, the results also showed that inhibition of SPRED1 counteracted the negative effects of miR‐KD‐IExos on the function of HUVECs. In conclusion, these results further indicated that exosomal miR‐126‐3p plays an important role in IExos‐mediated HUVEC activation by targeting SPRED1.

### 3.9 Exosomal miR‐126‐3p promotes proliferation, migration, and angiogenesis in HUVECs via SPRED1/Ras/Erk pathway

**FIGURE 8:**
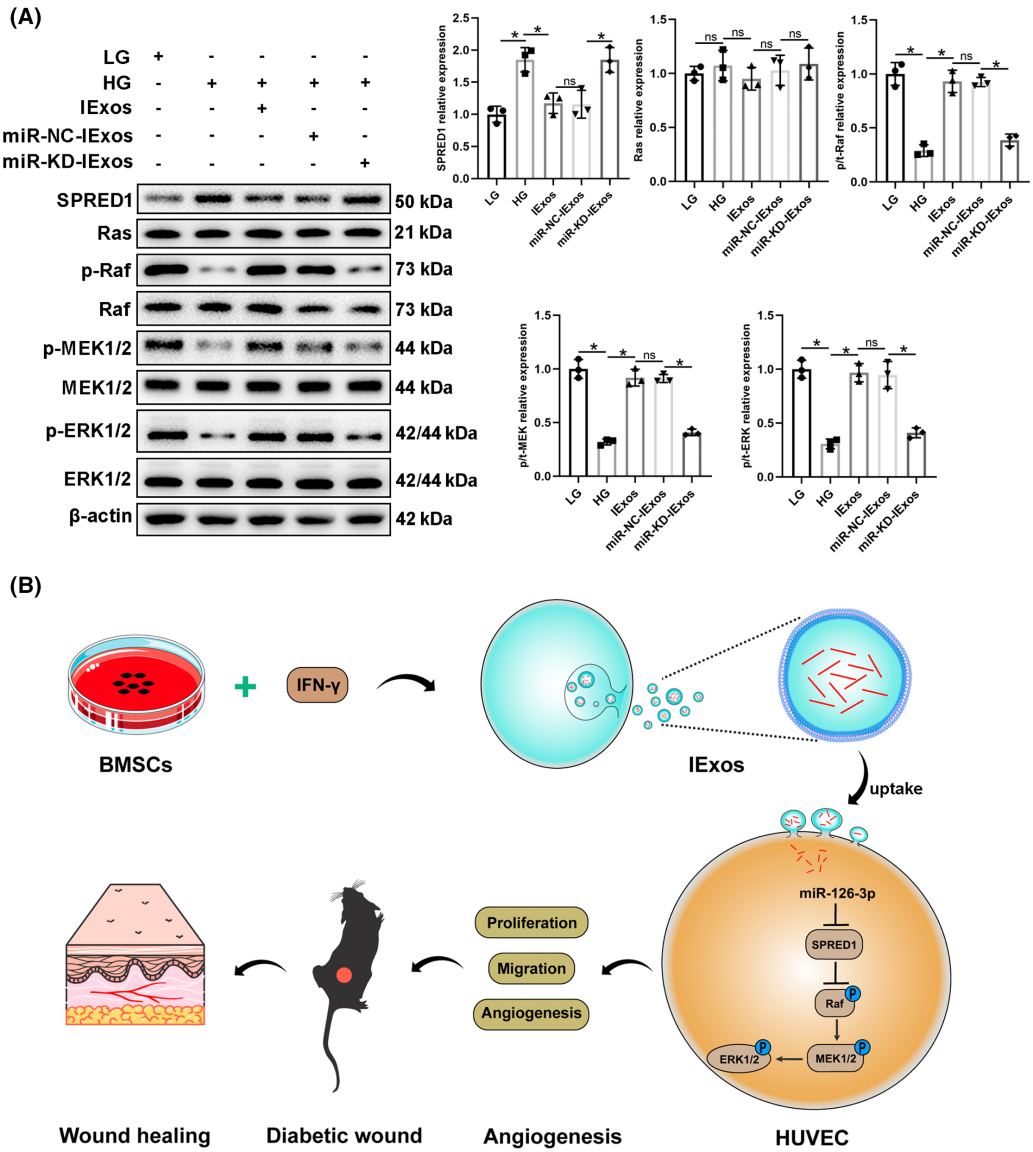
Exosomal miR‐126‐3p promotes proliferation, migration and angiogenesis in human umbilical vein endothelial cells (HUVECs) via the SPRED1/Ras/Erk pathway. (A) The protein levels were detected by western blot and quantified for SPRED1 and the downstream Ras/Erk signaling pathway in HUVECs treated with LG, HG + PBS, HG + IExos, HG + miR‐NC‐IExos, and HG + miR‐KD‐IExos. (B) Our results indicated that exosomes derived from IFN‐γ‐primed BMSCs can promote the function of HUVECs through regulating SPRED1/Ras/Erk axis. *p < .05 and *n* = 3 biological replicates. BMSCs, bone marrow mesenchymal stem cells; HG, high glucose; IExos, exosomes isolated from the supernatants of BMSCs with IFN‐γ treatment; IFN‐γ, interferon‐gamma; KD, knockdown; LG, low‐glucose; miRNA, microRNA; NExos, exosomes isolated from the supernatants of BMSCs without IFN‐γ treatment.

It has been reported that SPRED1 is an important molecule in the process of angiogenesis and can regulate several cellular activities, including motility, tube formation, and cell cycle through the Ras/Erk axis.^20^ Thus, we questioned whether exosomal miR‐126‐3p promotes the function of HUVECs via the SPRED1/Ras/Erk axis. HUVECs were treated with LG, HG, HG + IExos, HG + miR‐NC‐IExos, or HG + miR‐KD‐IExos, and western blotting was performed to detect the expression of SPRED1 and the main members of the Ras/Erk pathway. SPRED1 was significantly downregulated in cells treated with IExos and the expression of downstream molecules was increased. In contrast, the expression of SPRED1 was upregulated in the miR‐KD‐IExos group compared to that in the miR‐NC‐IExos group, and the downstream Ras/Erk pathway was suppressed (Figure 8A,B). To confirm the functional dependency between ERK signaling and IExos, we co‐cultured IExos and HUVECs treated with or without the specific inhibitor of ERK1/2 (PD98059). The results showed that PD98059 could remarkably suppressed the promotion of proliferation, migration, and angiogenesis brought by IExos (Figure S3). In conclusion, our results indicate that exosomes derived from IFN‐γ‐primed BMSCs can promote HUVEC function of HUVECs by regulating the SPRED1/Ras/Erk axis.

## 4 DISCUSSION

In the present study, we investigated the therapeutic effects of exosomes derived from BMSCs on diabetic wound healing. NExos treatment accelerated diabetic wound healing by promoting angiogenesis in vivo and in vitro, and IExos exhibited superior therapeutic efficiency. miR‐126‐3p was significantly upregulated in IExos, and exosomal miR‐126‐3p promoted angiogenesis and diabetic wound healing via transfer to HUVECs. Mechanistically, IFN‐γ‐pretreated MSCs secreted miR‐126‐3p‐enriched exosomes, which enhanced the function of HUVECs and promoted angiogenesis via the SPRED1/Ras/Erk pathway.

Exosomes derived from MSCs have been reported to show MSC‐like therapeutic effects in various disease^26^, ^27^, ^28^ and possess several advantages, including immune silencing, non‐oncogenicity, and high stability.^6^ In this context, exosome‐based treatment has attracted increasing interest, and its application in diabetic wound healing has been extensively studied over the past decade.^29^, ^30^ Consistent with previous studies, exosomes isolated from BMSCs showed therapeutic effects in diabetic wound healing in this study. Exosomes often possess different properties as the original MSCs change in response to external factors including cytokines and inflammation.^31^, ^32^ We examined the effect of exosomes isolated from IFN‐γ‐primed BMSCs and found that IExos exhibited higher therapeutic efficacy in diabetic wound healing. GW4869 was used to inhibit exosome release and corresponding CM of BMSCs with IFN‐γ treatment was collected. Interestingly, supernatants of IFN‐γ‐pretreated BMSCs can still significantly enhance the function of HUVECs (data not shown). We speculated that in addition to exosomes, there were also other proangiogenic components in the supernatants of IFN‐γ‐pretreated BMSCs. In conclusion, these results indicate that cytokine priming, especially IFN‐γ priming, may be a promising approach for increasing the therapeutic effects of BMSCs in diabetic wound healing.

Wound healing is a complex and dynamic process involving several biological processes and many types of cells, whereas impaired angiogenesis in diabetic patients influences the delivery of nutrition and oxygen to wound sites, which in turn affects diabetic wound healing.^33^ Angiogenesis is an important therapeutic target for wound healing and HUVECs are the important effector cells in skin wounds. Previous studies have reported that exosomes from different types of original MSCs can promote the function of HUVECs and facilitate wound healing. For example, Zhang et al found that exosomes isolated from adipose MSCs could improve the function of HUVECs in an HG environment.^34^ Extracellular vesicles from human umbilical cord MSCs were also found to facilitate diabetic wound healing by enhancing angiogenesis.^35^ Consistent with a previous study,^13^ exosomes derived from BMSCs were internalized by HUVECs and promoted angiogenesis in vitro and in vivo. Furthermore, exosomes derived from IFN‐γ‐primed BMSCs showed higher therapeutic efficiency in diabetic wound healing. These results indicate that IFN‐γ‐primed exosomes may be a potential alternative to HUVECs for treating diabetic wounds.

Exosomes can be transferred to recipient cells to mimic the function of their original cells by delivering proteins, RNAs, and DNAs.^36^ For instance, exosome‐cargoed microRNAs play a crucial role in exosome‐based therapy and are emerging as pivotal regulators of angiogenesis during wound closure.^9^ For example, Gondaliya et al found that treatment with miR‐155‐inhibitor‐loaded MSC‐derived exosomes led to enhanced collagen deposition, angiogenesis, and reepithelialization in diabetic wounds.^37^ Exosomes derived from atorvastatin‐pretreated MSCs accelerate diabetic wound repair by enhancing angiogenesis via the miR‐221‐3p/AKT/eNOS axis.^38^ However, the role of miRNAs in exosomes secreted from BMSCs after IFN‐γ treatment in diabetic wound repair remains unclear. A previous study performed miRNA sequencing and found that IFN‐γ pretreatment can induce a significant increase in five exosomal miRNAs.^19^ Among these differentially expressed exosomal miRNAs, miR‐126‐3p was found to be significantly upregulated in IExos. Considering that miR‐126‐3p has been reported to be secreted outside the cell in the form of exosomes to perform its function^39^, ^40^ and to regulate angiogenesis in several diseases,^20^, ^22^, ^41^, ^42^, ^43^ miR‐126‐3p was selected for further research in this study. Our results confirmed that miR‐126‐3p could be delivered from BMSCs to HUVECs in the form of exosomes. Furthermore, exosomal miR‐126‐3p can facilitate the function of HUVECs and angiogenesis under HG conditions both in vitro and in vivo.

The roles of SPRED proteins in signaling, development, and cancer are being increasingly recognized. SPRED1 has been recognized as an inhibitor of cell motility and Rho‐mediated actin reorganization, which are important for migration and proliferation.^44^ Several studies have confirmed that targeting SPRED1 may be a potential therapeutic approach for promoting angiogenesis in several vascular‐related diseases.^24^, ^45^, ^46^, ^47^ In diabetes, SPRED1 was also reported to be a therapeutic target in endothelial microparticle‐mediated vascular endothelial cell repair, and this mechanism was abrogated in glucose‐damaged endothelial microparticles.^48^ In the present study, SPRED1 was predicted to be a target gene of miR‐126‐3p, as confirmed by qRT‐PCR, western blotting, and luciferase reporter analyses. Loss of function experiments were performed, and the results showed that SPRED1 KD alleviated the negative effects of miR‐KD‐IExos on endothelial cell function. These results confirmed that exosomes derived from IFN‐γ‐primed BMSCs can promote endothelial cell function through exosomal miR‐126‐3p by targeting SPRED1.

## 5 CONCLUSION

In the current study, we aimed to confirm that exosomes derived from IFN‐γ‐primed BMSCs exhibit enhanced therapeutic efficiency in diabetic wound healing. IExos have been found to accelerate such healing by promoting the function of endothelial cells and improving angiogenesis. Mechanistically, IFN‐γ‐pretreated MSCs secreted miR‐126‐3p‐enriched exosomes, which enhanced the function of HUVECs and promoted angiogenesis via the SPRED1/Ras/Erk pathway. Our study represents an initial step toward the development of future therapies.

## AUTHOR CONTRIBUTIONS

Wen Lu and Xuan Du contributed equally to this work. Huijuan Li and Bimin Shi conceived and designed the study and acquired and interpreted the data. Wen Lu and Xuan Du were involved in acquisition and interpretation of data and drafting of the manuscript; Wen Lu and Shengyi Zou were involved in analysis and acquisition of data; Qionglei Fang and Mengjiao Wu were involved in critical revision of the manuscript for important intellectual content.

## FUNDING INFORMATION

This work was supported by the Key R&D Program of China (2019YFA0802400), Youth Science and Technology Foundation of Suzhou City of China (KJXW2022004), and Suzhou Science and Technology (SLT202008). [Correction added on 18 September 2023, after first online publication: the sequence of funding agencies has been rearranged.]

## CONFLICT OF INTEREST STATEMENT

The authors declare no conflicts of interest.

## Supporting information


**FIGURE S1.** Identification of bone marrow mesenchymal stem cells (BMSCs). (A) The isolated and cultured BMSCs appeared long spindle‐shaped. (B and C) Alkaline phosphatase staining and oil red O staining identified the adipogenic and osteogenic differentiation of BMSCs, respectively. (D) Flow cytometry analysis of BMSCs. *n* = 3 biological replicates.

**FIGURE S2.** Interferon‐γ pretreatment directly increase the expression of miR‐126‐3p in bone marrow mesenchymal stem cells (BMSCs), which resulted in the enrichment of exosomal miR‐126‐3p. The expression of miR‐126‐3p in BMSCs (A) under different conditions and in corresponding exosomes (B) were detected. *p < .05 and *n* = 3 biological replicates.

**FIGURE S3.** The functional dependency between ERK signaling and IExos was further confirmed. PD98059 could remarkably suppressed the promotion of proliferation (A and B), migration (C), and angiogenesis (D) brought by IExos. *p < .05 and *n* = 3 biological replicates.

**TABLE S1.** Primer sequences.

## Data Availability

The data presented in this study are available on request from the corresponding author.
