# Supplementary figures and images for: IFN‐γ enhances the therapeutic efficacy of MSCs‐derived exosome via miR‐126‐3p in diabetic wound healing by targeting SPRED1
Source: J Diabetes. 2023 Aug 30;16(1):e13465. doi: 10.1111/1753-0407.13465 (PMC10809290; doi:10.1111/1753-0407.13465)

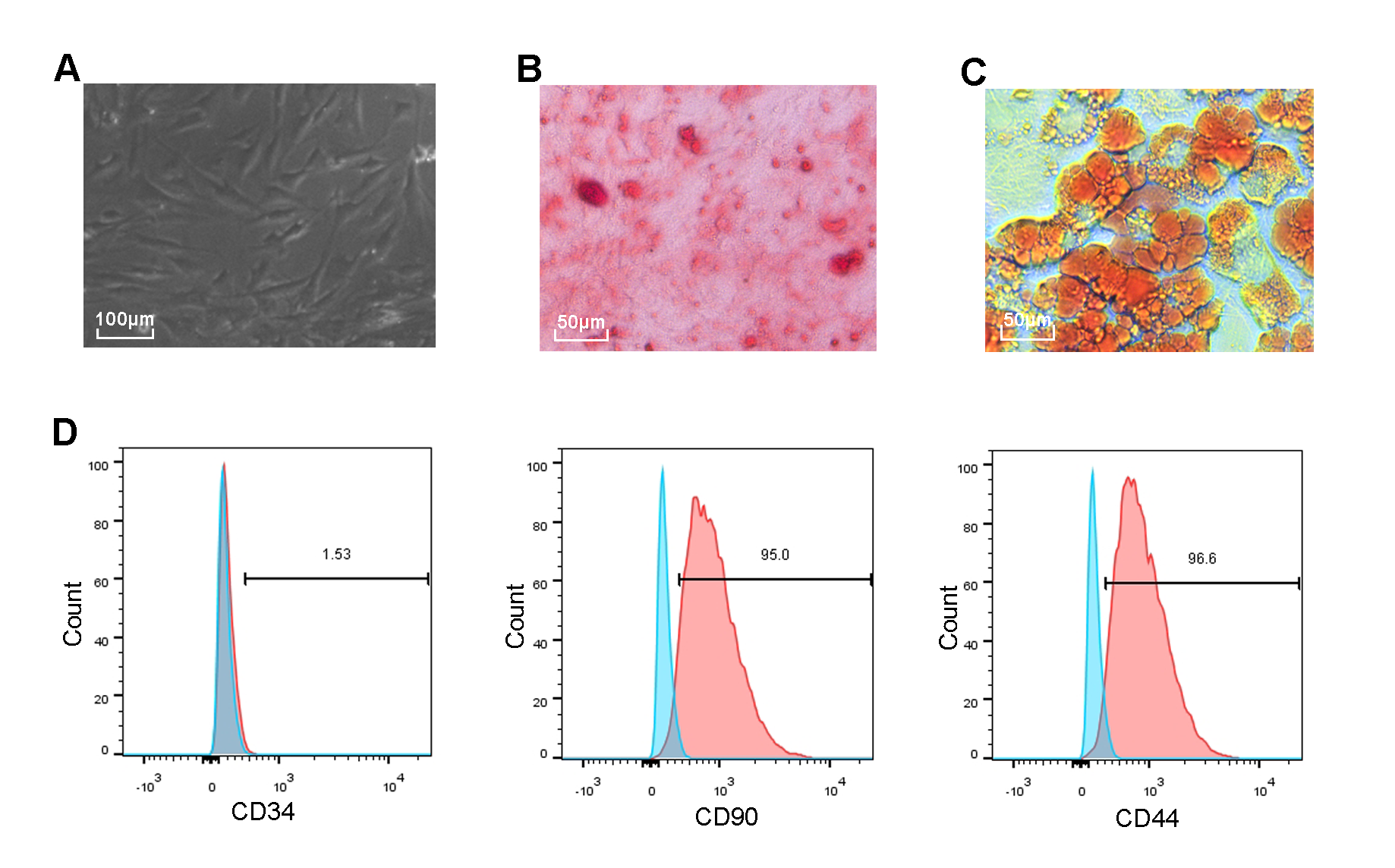

Supplement: Supplementary file 1 — FIGURE S1. Identification of bone marrow mesenchymal stem cells (BMSCs). (A) The isolated and cultured BMSCs appeared long spindle‐shaped. (B and C) Alkaline phosphatase staining and oil red O staining identified the adipogenic and osteogenic differentiation of BMSCs, respectively. (D) Flow cytometry analysis of BMSCs. n = 3 biological replicates. [file JDB-16-e13465-s001.tif]

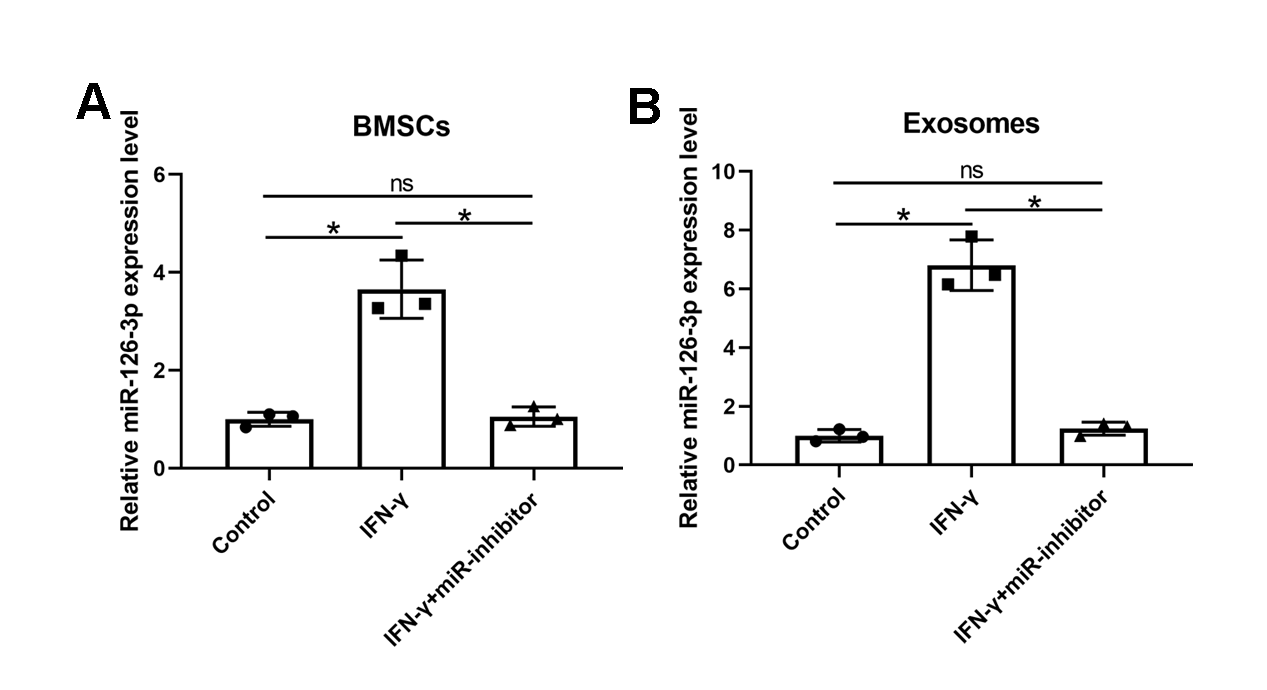

Supplement: Supplementary file 2 — FIGURE S2. Interferon‐γ pretreatment directly increase the expression of miR‐126‐3p in bone marrow mesenchymal stem cells (BMSCs), which resulted in the enrichment of exosomal miR‐126‐3p. The expression of miR‐126‐3p in BMSCs (A) under different conditions and in corresponding exosomes (B) were detected. *p < .05 and n = 3 biological replicates. [file JDB-16-e13465-s002.tif]

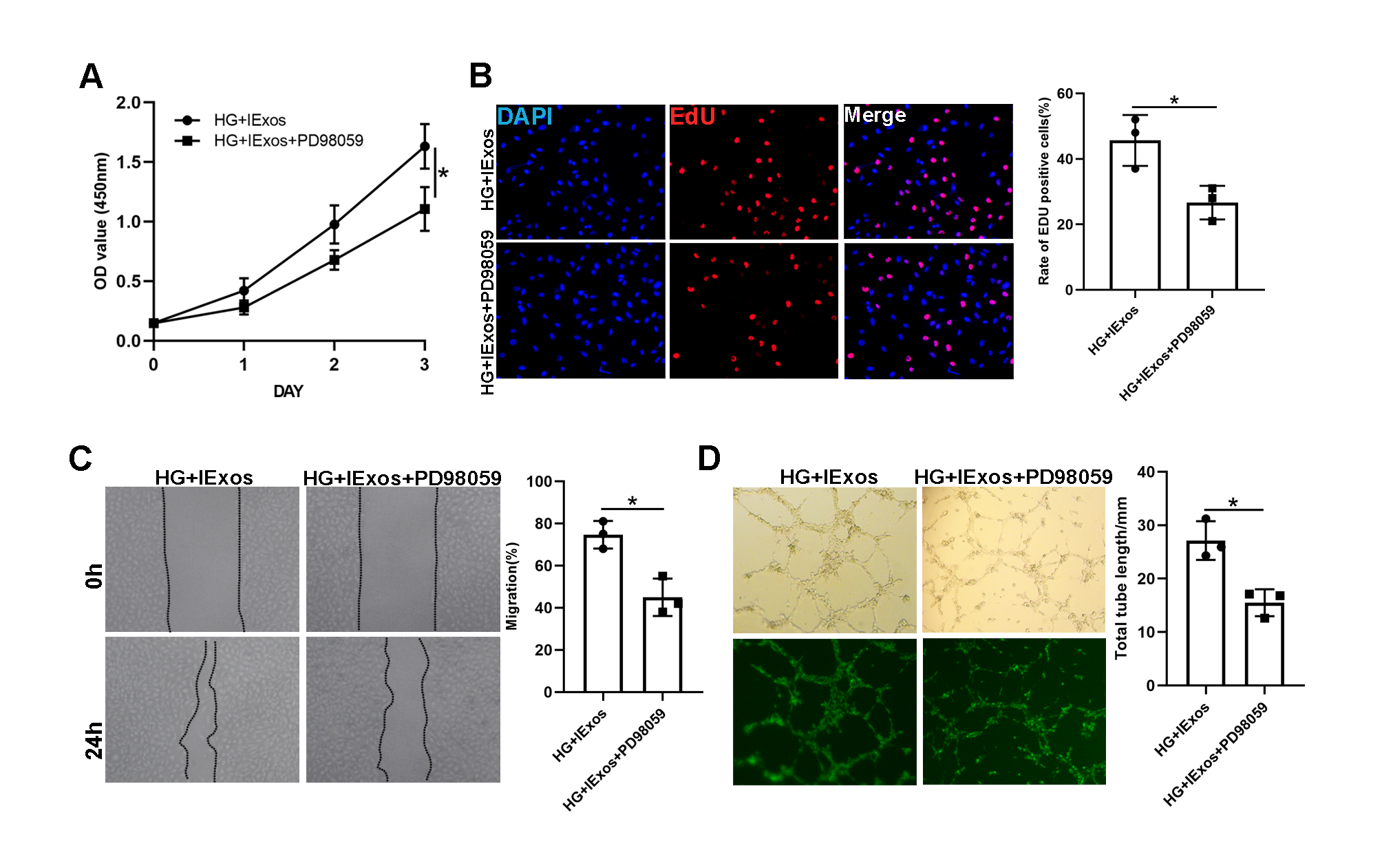

Supplement: Supplementary file 3 — FIGURE S3. The functional dependency between ERK signaling and IExos was further confirmed. PD98059 could remarkably suppressed the promotion of proliferation (A and B), migration (C), and angiogenesis (D) brought by IExos. *p < .05 and n = 3 biological replicates. [file JDB-16-e13465-s003.tif]
