# Supplementary material for: IFN‐γ enhances the therapeutic efficacy of MSCs‐derived exosome via miR‐126‐3p in diabetic wound healing by targeting SPRED1
Source: J Diabetes. 2023 Aug 30;16(1):e13465. doi: 10.1111/1753-0407.13465 (PMC10809290; doi:10.1111/1753-0407.13465)
Supplement: Supplementary file 4 — TABLE S1. Primer sequences. [file JDB-16-e13465-s004.docx]

| Table S1. Primer Sequences. | | |
| --- | --- | --- |
| ITGA6 | Forward | CTCTTCGGCTTCTCGCT |
|  | Reverse | CTGTTCTGTTCGCCCTCT |
| SPRED1 | Forward | CAGCCAGGCTTGGACATTCA |
|  | Reverse | TGGGACTTTAGGCTTCCACAT |
| IRS1 | Forward | CTCACCTTCAGCCACCA |
|  | Reverse | CGGACACGGAAGCACTA |
| CAMSAP1 | Forward | GTCCGCTATCGACGAGAGC |
|  | Reverse | GCCATCGACGTTACCTCCTTTA |
| β-actin | Forward | GTGAAAAGATGACCCAGATCAT |
|  | Reverse | GCTTCTCTTTGATGTCACGCACGAT |
